# Deep layer neurons in the rat medial entorhinal cortex fire sparsely irrespective of spatial novelty

**DOI:** 10.3389/fncir.2014.00074

**Published:** 2014-07-11

**Authors:** Andrea Burgalossi, Moritz von Heimendahl, Michael Brecht

**Affiliations:** ^1^Bernstein Center for Computational Neuroscience, Humboldt University of BerlinBerlin, Germany; ^2^Werner Reichardt Centre for Integrative Neuroscience, University of TübingenTübingen, Germany

**Keywords:** sparse coding, medial entorhinal cortex, novelty, rat, spatial

## Abstract

Extracellular recordings in medial entorhinal cortex have revealed the existence of spatially-modulated firing patterns, which are thought to contribute to a cognitive map of external space. Previous work indicated that during exploration of novel environments, spiking activity in deep entorhinal layers is much sparser than in superficial layers. In the present report, we ask whether this laminar activity profile is a consequence of environmental novelty. We report on a large dataset of juxtacellularly-recorded neurons (*n* = 70) whose spiking activity was monitored while rats explored either a novel or a familiar environment, or both within the same session. Irrespective of previous knowledge of the environment, deep layer activity was very low during exploration (median firing rate 0.4 Hz for non-silent cells), with a large fraction of silent cells (*n* = 19 of a total 37), while superficial layer activity was several times higher (median firing rate 2.4 Hz; *n* = 33). The persistence of laminar differences in firing activity both under environmental novelty and familiarity, and even in head-restrained stationary animals, suggests that sparse coding might be a constitutive feature of deep entorhinal layers.

## Introduction

The medial entorhinal cortex (MEC) is a key structure involved in processing of spatial information (Fyhn et al., 2004; Hafting et al., 2005; Derdikman and Moser, 2010). Superficial (L2-3) and deep layers (L5-6) of the MEC show clear differences in anatomical connectivity, as well as intrinsic and functional neuronal properties (Sargolini et al., 2006; Canto and Witter, 2012). While the superficial layers of the MEC are the main recipient of processed sensory information and give rise to the major projection to the hippocampal formation via the perforant path, the deep layers receive feedback from the hippocampal subfields CA1 and subiculum. Although there are many exceptions from this simplified picture (Van Strien et al., 2009), the layer differences seem to predict distinct functional roles of entorhinal layers in spatial processing.

In a recent study (Burgalossi et al., 2011), we employed the juxtacellular recording method (Pinault, 1996) to sample individual MEC neurons in behaving animals. An advantage of the juxtacellular recording technique over conventional extracellular methods is that neurons can be identified irrespective of their spiking activity (Herfst et al., 2012). Neurons contributing very few spikes during the recording session, and even silent cells can be reliably recorded (Zhang and Deschênes, 1997). Sampling of individual neurons by juxtacellular method revealed strong laminar differences in firing rates in animals exploring novel environments: deep layer neurons displayed much lower rates (all below 1 Hz, with nearly half of them silent) than superficially recorded cells (Burgalossi et al., 2011). While layer differences might be expected due to the marked differences in anatomical connectivity and intrinsic neuronal properties, the reason for the reported low rates in deep layers remains unclear.

Spatial novelty appears to be one important trigger for the creation of new spatial maps in the rodent hippocampus (Muller and Kubie, 1987), and the expansion (Barry et al., 2012) and re-alignment of grid maps in the MEC modules (Fyhn et al., 2007). Furthermore, novelty can modulate theta (4–12 Hz) oscillatory dynamics (Wells et al., 2013) and firing activity of neurons in the hippocampal circuit (Nitz and McNaughton, 2004; Larkin et al., 2014). We therefore asked whether environmental novelty contributes to the low activity of deep layer neurons. To this end, we juxtacellularly recorded single neurons while rats explored either a novel or a familiar environment, or both within the same session. The present study, which is based on a large dataset of juxtacellularly recorded neurons (*n* = 70) indicates that firing activity is not significantly modulated by environmental novelty, but the activity's laminar differences might rather be a constitutive feature of entorhinal circuits.

## Materials and methods

### Recording apparatus and behavioral training

Two types of arenas were used in this study: an O-maze (*n* = 45 experiments) and a two-compartment maze (*n* = 25 experiments). The O-maze (60 × 120 cm) had 22-cm high inner and outer walls, with a 12-cm wide path in between. A small subset of neurons from our previous study (Burgalossi et al., 2011) were recorded in a smaller O-maze (40 × 80 cm, *n* = 6). The two-compartment maze was comprised of a novel and a familiar compartment. The novel one was square-shaped (55 × 55 cm) and had 80 cm high walls, whose inner face was covered with black and white stripes of tape, and a soft texture was added to the floor (a 0.5-cm thick sponge layer covered with black insulating adhesive foil). The familiar compartment was a rectangular arena (81 × 25 cm) with 21-cm high walls. The familiar and novel compartment communicated via a gate, which could be manually opened.

For recordings in familiar environments (either O-maze or two-compartment maze) the rats were placed in the arena for 2–4 sessions per day (lasting between 15 min and 1 h each) for 3–7 days. In the two-compartment maze, in order to limit use of distal visual cues, the novel compartment was dimly illuminated from the top. During habituation in the familiar compartment, the access gate to the novel compartment was always closed. These measures were taken to ensure complete novelty of the novel compartment in the two-compartment maze, as opposed to a mixed situation where only local cues are novel, which leads to different cognitive processing (Leutgeb et al., 2005). After each session, the floor of the mazes was cleaned with ethanol.

### *In-vivo* juxtacellular recordings

Juxtacellular recordings in freely-moving animals were obtained according to previously published procedures (Burgalossi et al., 2011; Herfst et al., 2012). For recordings in novel environments, naïve Wistar rats were initially anesthetized with a mixture of medetomidine (225 μg/kg), midazolam (6 mg/kg), and fentanyl (7.5 μg/kg). After a juxtacellular recording was obtained and mechanically stabilized (Herfst et al., 2012), the rat was placed in the novel environment and the anesthetics were reversed by a fast-acting mix of antagonists (antipamezole, 1 mg/kg; flumazenil, 600 μg/kg; naloxone, 180 μg/kg; Lee et al., 2006; Burgalossi et al., 2011; Herfst et al., 2012) and the spiking activity of the neuron monitored while the rat explored the environment.

For recordings in familiar environments, animals were habituated to the O-maze for 3–7 days (2–4 sessions per day, of 15 min–1 h duration each). On the day of the experiment, animals were anesthetized with the antagonizable anesthetic (see above) and the recordings performed as described above.

For recordings in the two-compartment maze (*n* = 17), animals were pre-implanted under ketamine/xylazine anesthesia (intraperitoneal doses of 100 and 10 mg/kg, respectively) according to previously published procedures (Houweling and Brecht, 2008; Herfst et al., 2012). After a recovery period (typically 2–3 days) animals were habituated to the familiar compartment of the two-compartment maze for 3–7 days (2–4 sessions per day, of 15 min–1 h duration each). Habituation was performed either before and after implantation, or only after implantation. On the day of the experiment, animals were anesthetized with the antagonizable anesthetic (see above) and the recordings performed as described above.

One cell per animal was recorded. In all cases, recording sites could be clearly identified by biocytin/neurobiotin spillover at the ejection site, thereby providing unequivocal assignment of the recording layer even when cell recovery failed.

Juxtacellular recordings in drug-free, head-fixed rats were performed as previously described (Houweling and Brecht, 2008; Doron et al., 2014). Briefly, animals were pre-implanted with a metal post and a recording chamber under ketamine/xylazine anesthesia, and a craniotomy was performed at the coordinates for targeting MEC (Burgalossi et al., 2011). The craniotomy was then closed with silicone (Kwik-Cast, World Precision Instruments). After a recovery period (2–3 days), animals were slowly habituated to the head-fixation. After successful habituation (3–7 days) animals were head-fixed, the silicone plug was removed and the craniotomy carefully cleaned. Before juxtacellular recordings, mapping experiments with low-resistance electrodes (0.5–1 MΩ) were performed to estimate the recording depth of the entorhinal layers, based on known electrophysiological features of the entorhinal laminar structure (Quilichini et al., 2010). Juxtacellular recordings in head-fixed animals (*n* = 49) were assigned to superficial and deep layers based on (1) cortical depth and mapping experiments and (2) morphological identification of a subset of the recorded neurons (*n* = 9), which confirmed the layer assessment. Recordings and habituation sessions were performed under dim ambient illumination.

The juxtacellular signal was amplified by an ELC Ultra miniature headstage (NPI Electronic), and an ELC-03XS amplifier (NPI Electronic), sampled at 20–50 kHz by a LIH 1600 data-acquisition interface (HEKA Electronic) under the control of PatchMaster 2.20 software (HEKA Electronic). The location of the animal was tracked at 25 Hz by the Digital Lynx video-tracking system (Neuralynx) using two LEDs (red and blue) mounted on the rat's head. All experimental procedures were performed according to German guidelines on animal welfare under the supervision of local ethics committees.

### Histological analysis

At the end of each recording, the animal was injected with an overdose of ketamine or urethane and quickly perfused transcardially with 0.1 M phosphate-buffered saline followed by a 4% paraformaldehyde solution. To reveal the morphology of juxtacellularly labeled cells, 100–150 μm thick brain slices were processed with the avidin-biotin-peroxidase method as described previously (Lee et al., 2006, 2009; Epsztein et al., 2010). Neurons were manually reconstructed with Neurolucida software (MBF Bioscience) and displayed as a two-dimensional projection.

### Data analysis

#### Behavior

The position of the rat was defined as the midpoint between the two head-mounted LEDs. To determine running speed, the rat's head positions was first smoothed with a square window of length 600 ms, to decrease the impact of jerky head motion.

Center field avoidance in the square, novel part of the two-compartment maze was quantified by measuring the relative occupancy in an inner square half the side length of the full square. This inner square occupied one quarter of the total area, and therefore center field avoidance was assessed by comparing its relative occupancy to 25%.

Preference for the familiar part of the two-compartment maze was measured as the share of time spent in the familiar compartment, counting from the moment the rat entered it first. To avoid bias, a characteristic time *T* was estimated as the time required to traverse the familiar part of the maze, *T* = *L/v*, with *L* the length of the maze and *v* the rat-specific average speed. Values of *T* ranged from 9 to 53 s. *T* was used for two corrective steps: Immediately after the first entry into the familiar compartment, a period of *T* was ignored for analysis. This was done to eliminate bias because initially, the rat had to be in the familiar compartment, by definition. Second, an adjusted share of time spent in the familiar maze was calculated as

$$s_{\text{adj}}=(t_{\text{fam}}+T/2)/(t_{\text{total}}+T),$$

with *t*_fam_ the time spent in the familiar compartment, *t*_total_ the total time, *T* the characteristic time and *s*_adj_ the adjusted familiar share. The adjustment with *T*/2 and *T* is a standard statistical measure to avoid edge problems and give appropriate weight to null and full shares. The distribution across rats of familiar shares was guessed to be logit-normal, which was verified by logit-transforming all *s*_adj_ and testing for normality (Lilliefors test for normality, *p* = 0.5). Then, the logit-transformed values were tested for a mean of 0 (because logit of the critical value of 50% is 0) using a *t*-test.

#### Physiology

A fraction of recordings performed in novel environments (*n* = 31) have been published elsewhere (Burgalossi et al., 2011). For spike analysis, juxtacellular traces were high-pass filtered at 100 Hz, and a three-dimensional analysis using time and the first two principal components of the waveform was performed to visualize and assess the stability of spikes amplitude over time, and to isolate spikes from recording artifacts. For most recordings (including silent cells) the electrode resistance was monitored by small (<1 nA) hyperpolarizing current pulses delivered every 20–30 s. Loss of the juxtacellular configuration was signaled by a sudden loss of the spike signals (for spiking cells) and/or a concomitant drop in the electrode tip resistance. In a large fraction of recordings from silent cells (70%; *n* = 12/17) the neurons were fired at the end of the recording session by positive current injections to directly confirm the presence of the cell.

For the calculation of firing rates (including the identification of silent cells), only epochs with speeds greater than 1 cm/s were considered. An exception was the inclusion criterion for the paired test used in the two-compartment maze, where cells were included if only they fired a single spike ever, in either compartment.

Firing rate distributions were tested for bimodality on a logarithmic scale. To resolve the rates without any recorded spike, a virtual spike was added to all counts. This is a standard measure to resolve singularity problems and, at the same time, give appropriate weight to zero-count samples; e.g., a recording without observed spikes of 10 s is represented as a rate of 1 * 10^−1^ Hz, but 10 min without a spike as 0.2 * 10^−2^ Hz, effectively a “stronger” zero.

The firing rate distribution was tested for bimodality by calculation of the bimodality coefficient described by Pfister et al. (2013).

$$\text{BC}=\frac{m_{3}^{2}+1}{m_{4}+3\cdot \frac{{\left(n-1\right)}^{2}}{\left(n-2)(n-3\right)}},$$

with *m*_3_ referring to the skewness of the distribution, *m*_4_ its excess kurtosis and *n* the sample size. Values of BC greater than 0.55 are considered an indication of bimodality (Pfister et al., 2013). As another independent measure, Hartigan's dip test was performed (Hartigan and Hartigan, 1985). To test for significance, the dip value was calculated on 1000 random samples of uniform distributions of sample size equal to the real data, and the real dip value ranked in the random distribution.

## Results

### Deep layer neurons fire at low rates irrespective of spatial experience

Juxtacellular recordings in freely-moving animals were obtained according to previously published procedures (Burgalossi et al., 2011; Herfst et al., 2012). Briefly, animals were anesthetized with an antagonizable anesthetic mix (Lee et al., 2006, 2009; Burgalossi et al., 2011; see Materials and Methods). After a juxtacellular recording was obtained, it was mechanically stabilized (Herfst et al., 2012) and the neuron labeled. Animals were woken up by injection of fast-acting antagonists (see Materials and Methods) and single cell spiking activity was monitored while rats explored an O-maze, either for the first time (“novel”) or after several days of habituation (“familiar”) (median recording duration = 198 s, interquartile range 114–370 s).

**Figure 1:**
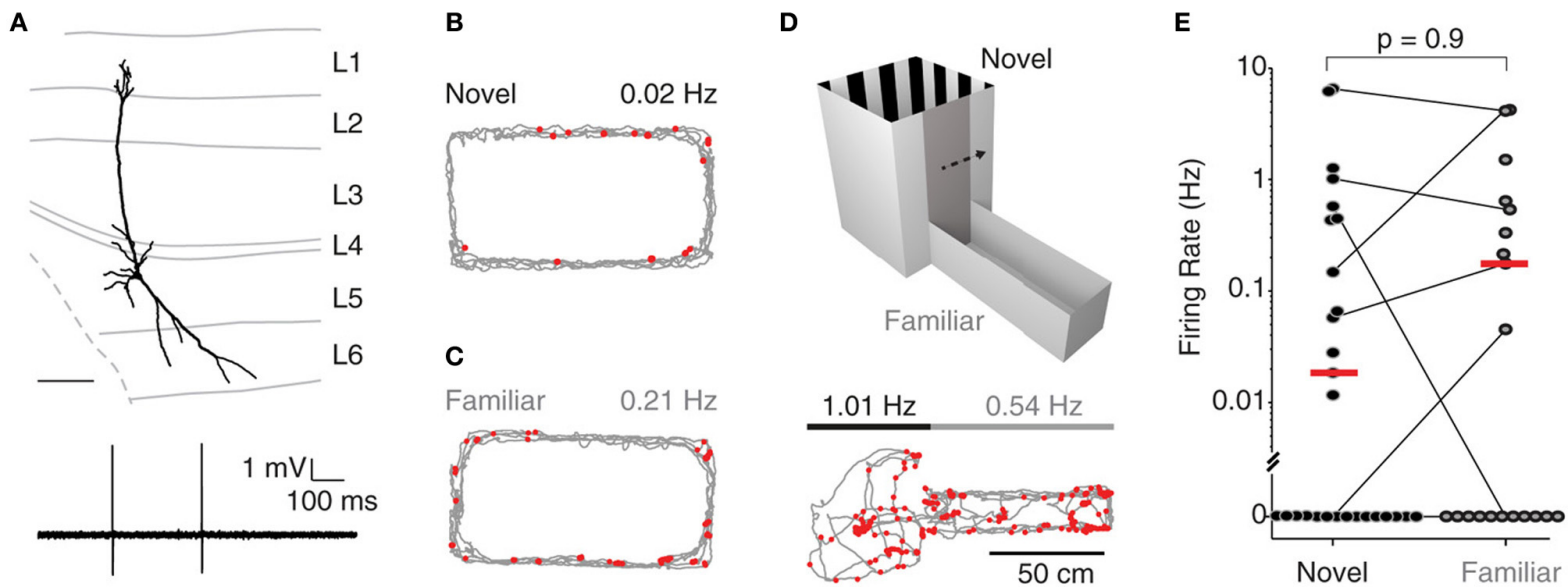
**Deep layer neurons in MEC fire at low rates irrespective of spatial experience. (A)** Top, reconstruction of the dendritic morphology of the L5 neuron of MEC recorded during exploration of a two-compartment maze (recording shown in **D**). Scale bar = 200 μm. Bottom, representative spike trace recorded during freely-moving behavior. **(B)** Firing locations of spikes (red) and trajectory of the rat (gray) during running in a novel environment (O-maze). Average firing rate is indicated. **(C)** Same as in **(B)**, but for a familiar environment. **(D)** Top: The two-compartment maze used for the combined novel/familiar experiments. Bottom: as in **(B)**, but for a recording in the two-compartment maze (square compartment, novel; rectangular compartment, familiar). Average firing rates in novel and familiar compartments are indicated. **(E)** Average firing rates of all juxtacellularly recorded neurons in novel and familiar environments. Horizontal lines connect recordings from the two-compartment maze, where novel and familiar compartments where sampled within the same recording session. Red lines indicate medians.

Recordings were targeted to deep layers (L5-6) of MEC; Figure 1A shows a representative L5 pyramidal neuron, recorded and identified in a freely-moving animal. During exploration of a novel environment, juxtacellularly recorded neurons in deep layers displayed very low levels of activity (median 0.01 Hz, *n* = 11; see Figure 1B for an example), with 5 of 11 neurons being completely silent (see also Burgalossi et al., 2011). To test whether the low firing activity in deep layers was attributable to the novelty of the environment, we performed nine additional juxtacellular recordings from rats which were habituated to the same O-maze prior to the recording session (see Materials and Methods). Even under these conditions, we observed a large fraction of silent cells (5/9; see Figure 1C for an example) and a similar distribution of firing rates (novel: median 0.01 Hz, range 0.0–1.3 Hz; familiar: median 0.0 Hz, range 0.0–1.5 Hz; *p* = 0.9, Wilcoxon rank-sum test, two-tailed).

To more directly assess the effect of spatial experience on the firing activity of deep layer neurons, we performed an additional set of experiments (*n* = 17) where single neuron spiking activity was recorded while rats explored both a novel and a familiar environment in a two-compartment maze (Figure 1D). In 11 of the 17 recordings, the animal explored both environments; in seven of these, the cell was active in at least one of the two environments. The two-compartment maze consisted of a square maze (“novel compartment”) connected to a rectangular maze (“familiar compartment”) separated by a sliding door (see Materials and Methods). Rats were first habituated to the familiar compartment of the maze for several days, and then a juxtacellular recording was obtained from a deep layer neuron and the rat woken up in the novel compartment. After exploring the novel compartment (range of traveled distances: 131–523 cm; *n* = 7) the gate was opened and the rat allowed to transition to the familiar compartment. Figure 1D shows a representative recording from a pyramidal neuron (reconstruction shown in Figure 1A) which was similarly active in both compartments. At the population level, there was no significant difference between firing rates in the novel and the familiar compartment (Figure 1E; median rates = 0.15 Hz (novel) and 0.17 Hz (familiar), *p* = 0.8, *n* = 7; Wilcoxon signed rank test, two-tailed). This remained true when all data from the O-mazes and the two-compartment maze were analyzed jointly (novel: median 0.00 Hz, range 0.0–6.5 Hz, *n* = 28; familiar: median 0.0 Hz, range 0.0–4.3 Hz, *n* = 20; medians not significantly different, *p* = 0.9, Wilcoxon rank-sum test, two-tailed). These data indicate that at the population level, spiking activity in deep layers of MEC is not significantly modulated by spatial experience.

### Superficial layer neurons fire at higher rates irrespective of spatial experience

**Figure 2:**
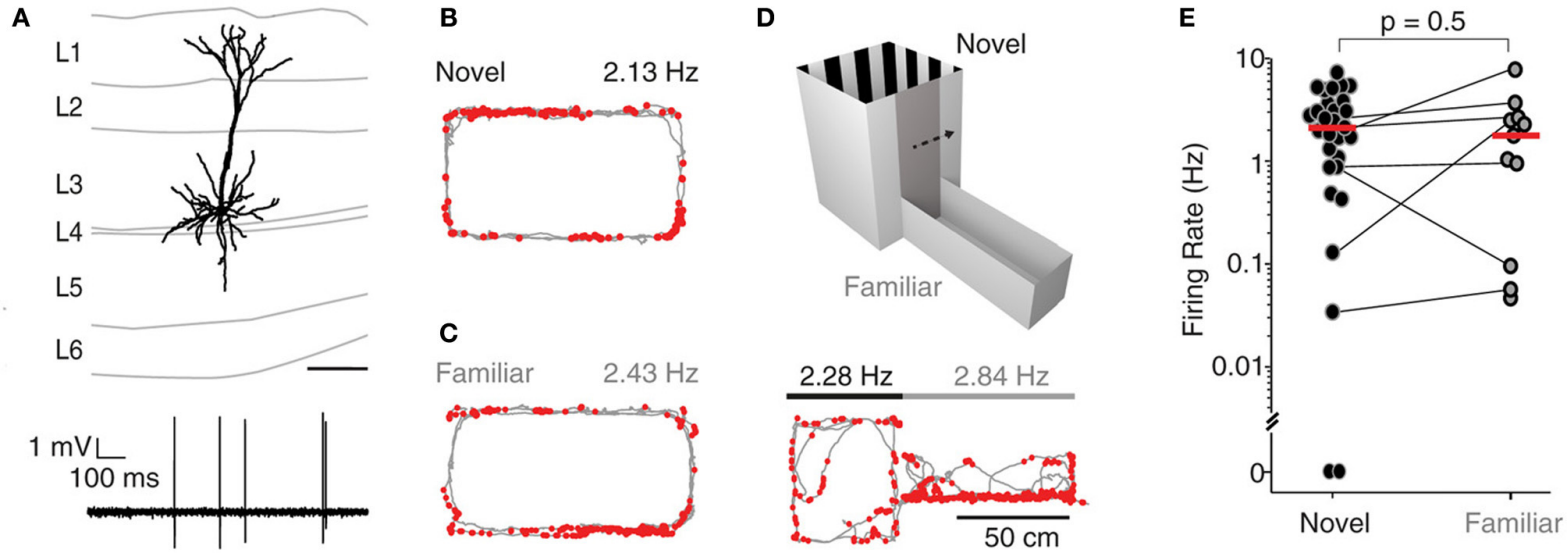
**Superficial layer neurons in MEC fire at higher rates irrespective of spatial experience**. **(A)** Top, reconstruction of the dendritic morphology of the L3 neuron of MEC recorded during exploration of a familiar O-maze [recording shown in **(C)**]. Bottom, representative spike trace. Scale bar = 200 during freely-moving behavior. **(B)** Firing locations of spikes (red) and trajectory of the rat (gray) during running in a novel environment (O- maze). Average firing rate is indicated. **(C)** Same as in **(B)**, but for a familiar environment. **(D)** Top: the two-compartment maze used for the combined novel/familiar experiments. Bottom: as in **(B)**, but for a recording in the two-compartment maze (square compartment, novel; rectangular compartment, familiar). Average firing rates in novel and familiar compartments are indicated. **(E)** Average firing rates of all juxtacellularly recorded neurons in novel and familiar environments. Horizontal lines connect recordings from the two-compartment maze, where novel and familiar compartments where sampled within the same recording session. Red lines indicate medians.

A subset of recordings (*n* = 33) was targeted to the superficial layers (L2-3) of MEC, and spiking activity from single neurons was monitored while rats explored either a novel, a familiar or a two-compartment maze. Figure 2A shows a representative L3 pyramidal neuron, recorded and identified in a freely-moving animal. During exploration of an O-maze novel to the rat, neurons juxtacellularly recorded from superficial layers displayed higher levels of activity compared to deep layer neurons, with only 1 of 21 neurons being silent (median 3.2 Hz, *n* = 21; see Figure 2B for an example; inter-layer comparison statistics in the next section). To test whether spatial experience has an impact on the activity of superficial layer neurons, we also recorded single neurons while rats explored a familiar O-maze (*n* = 4; Figure 2C, corresponding reconstruction shown in Figure 2A), and a two-compartment maze (*n* = 7; Figure 2D). Firing rates in novel and familiar environments did not differ significantly (median firing rate novel = 2.3 Hz, *n* = 29; median firing rate familiar = 1.9 Hz, *n* = 11; *p* = 0.5, Wilcoxon rank-sum test, Figure 2E), suggesting that superficial layer activity is not significantly modulated by spatial experience.

The absence of any difference in firing rates under manipulation of novelty raises the concern that the familiarity may not have been detected by the animals. To address this, the behavior was analyzed for novelty-driven patterns. Indeed, in the O-maze experiments, rats were more active in novel environments than in familiar ones (median of rats' average speeds: novel, 4.6 cm/s; familiar, 3.1 cm/s; *p* = 0.035, Wilcoxon rank-sum test, one-tailed; see Figure S1A), consistent with novelty-driven exploration. Similarly, in the two-compartment maze, rats were more active in the novel part (median of rats' average speeds: novel, 4.4 cm/s; familiar, 3.7 cm/s; *p* = 0.037, Wilcoxon rank-sum test, one-tailed; Figure S1B). Consistent with well described neophobic behaviors in rodents (Barnett, 1958), our rats showed open field avoidance in the novel part of the two-compartment maze (median center field occupancy 18%, less than the uniformly expected 25%, *p* = 0.048, sign test, one-tailed; Figure S1C) and once in the familiar, they avoided return to the novel environment (average share of time spent in the familiar compartment: 0.82, greater than 0.5 with *p* = 7.10^−7^, *t*-test, two-sided; Figure S1D, see Materials and Methods for details).

### Layer is a strong determinant of average firing rate

**Figure 3:**
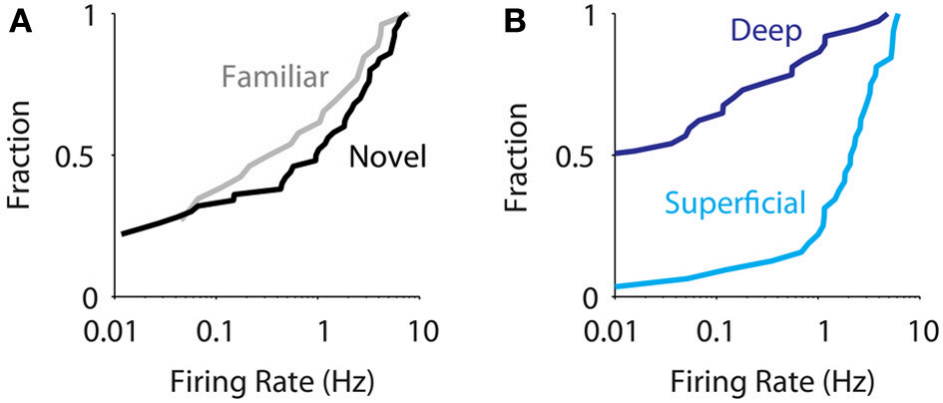
**Layer is a strong determinant of average firing rate in MEC**. **(A)** Cumulative distribution of average firing rates for all juxtacellularly recorded neurons recorded in novel (black, *n* = 57) and familiar environments (gray, *n* = 31). The two distributions are not statistically different [median for novel 0.54 (*n* = 57), familiar 0.17 (*n* = 31); *p* = 0.4, Wilcoxon rank-sum test]. **(B)** Cumulative distribution of average firing rates for all juxtacellularly recorded neurons recorded in superficial (light blue, *n* = 33) vs. deep layers (dark blue, *n* = 37), irrespective of spatial experience. The two distributions are statistically different (median for superficial 2.3, deep 0.0, *p* = 1.4 10^−7^, Wilcoxon rank-sum test).

Altogether, we juxtacellularly sampled the activity of 33 and 37 neurons in superficial and deep layers of MEC, respectively. At the population level (*n* = 70) we did not observe an effect of spatial experience in modulating the activity of MEC neurons [Figure 3A, median for novel 0.54 Hz (*n* = 57), familiar 0.17 Hz (*n* = 31); *p* = 0.4, Wilcoxon rank-sum test] in agreement with data obtained from extracellular recordings (Barry et al., 2012). On the other hand, we observed a robust difference in firing activity between superficial and deep neurons (Figure 3B, median for superficial 2.4 Hz, deep 0.0 Hz, *p* = 10^−7^, Wilcoxon rank-sum test). This was true for the new dataset alone [superficial layers: median 1.5 (*n* = 12), deep layers: median 0.00 (*n* = 27), *p* = 0.006, Wilcoxon rank-sum test, two-tailed], in line with the previously published data (Burgalossi et al., 2011). Within the deep and superficial layer groups, there were no significant differences either in firing rates [layers 2 vs. 3: medians 2.0 (*n* = 14) and 2.8 (*n* = 18), respectively, *p* = 0.3; layers 5 vs. 6: medians 0.0 (*n* = 26) and 0.14 (*n* = 11), *p* = 0.33; Wilcoxon rank-sum tests] or in the fraction of silent cells [layers 2 vs. 3: silent cells 1/14 and 1/18, respectively, *p* = 1; layers 5 vs. 6, silent cells 14/26 and 5/11, *p* = 0.7, Fisher exact tests]. Notably, the strong differences in firing activity between superficial and deep layer neurons were observed also in drug-free, awake, head-fixed animals (see Materials and Methods; median firing rates: superficial, 2.0 Hz, *n* = 28; deep, 0.1 Hz, *n* = 21; *p* = 9.10^−8^, Wilcoxon rank-sum test), suggesting they occur even in the absence of locomotor activity.

A difference in median firing rate between two populations can have different structures. Inspection of the firing rate histograms for all cells (Figure S2A) as well as by layer (Figure S2B) suggested that the median difference between deep and superficial cells may be due to different mixtures of two distributions: one with mainly silent and one with active neurons. We therefore tested for bimodality by applying two established criteria. While the bimodality coefficient (Pfister et al., 2013) was found to be 0.70 (greater than the conventional cut-off of 0.55, and therefore considered bimodal), Hartigan's dip test (Hartigan and Hartigan, 1985) did not indicate significant bimodality (dip = 0.029, *p* = 0.97, bootstrap test). Also, firing rate distributions differed between deep and superficial layers even considering non-silent cells only (superficial layers, median firing rate = 2.6 Hz, *n* = 31; deep layers, median firing rate = 0.38 Hz, *n* = 18, *p* = 0.0007, Wilcoxon rank-sum test). We therefore conclude that there is no conclusive evidence for a bimodal distribution of firing rates in MEC.

Irrespective of the statistically rigorous concept of bimodality, one striking feature of the firing rate distributions are the contrasting shares of silent cells between superficial and deep layers (see above), which can be interpreted as a measure of population sparseness (Willmore and Tolhurst, 2001). Deep layer neurons have a higher population sparseness than superficial layer neurons, and sparseness is unaffected by familiarity (number of silent cells; deep layers: familiar 11/20, novel 15/28, *p* = 1; superficial layers: familiar 0/11, novel 2/29, *p* = 1, both Fisher exact tests). Together, these findings indicate that population sparseness and neuronal firing rates in MEC differ strongly by layer but are not affected by environmental novelty.

## Discussion

It has been suggested that the cerebral cortex employs a sparse code for encoding information (Field, 1994). This has, indeed, been observed in sensory cortices (Huber et al., 2008; Wolfe et al., 2010; Barth and Poulet, 2012) and, notably, in the hippocampus (Thompson and Best, 1989). The present study indicates that environments are differentially encoded across layers of MEC, irrespective of their familiarity. While superficial neurons were equally active in both conditions (Figures 2, 3), deep layer neurons were mostly inactive, irrespective of previous knowledge of the environment (Figures 1, 3). These findings suggest that encoding of spatial information by deep entorhinal layers is sparser than previously assumed based on extracellular sampling of neuronal spiking activity (Sargolini et al., 2006). So far, layer specific differences in MEC have been reported with respect to neuronal functional properties: In rats exploring familiar environments, pure grid responses were abundant in layer 2, while conjunctive, head-direction and grid cells were present in different proportions across layers 3–6 (Sargolini et al., 2006). Here we extend previous observations on laminar activity differences in MEC (Burgalossi et al., 2011) by showing that they persist under environmental familiarity.

The juxtacellular method provides unique advantages over classical extracellular recording techniques. First, it allows identification of the recorded unit and unequivocal laminar localization of the recording site (Herfst et al., 2012; see Materials and Methods). Second, it provides more realistic estimates of layer activity, since neurons can be sampled irrespective of their spiking activity (Zhang and Deschênes, 1997; Herfst et al., 2012). Silent cells, or cells which contribute very few spikes during the behavioral recording session, cannot be easily detected with extracellular recording methods, and their presence can only be indirectly inferred (Thompson and Best, 1989; Neunuebel and Knierim, 2012).

The anesthesia/wake-up protocol used in the present study has limitations, which might potentially have occluded a physiological novelty effect. Although the behavior of the rats appeared to be largely unaffected (see below), potential residual effects of the anesthetics cannot be ruled out, as acknowledged previously (Burgalossi et al., 2011; Herfst et al., 2012). However, the concerns of anesthesia side-effects are diminished by the fact that laminar differences persisted even in awake, drug-free animals. Another concern is that exposure to a novel context occurred immediately after wake up, which might be source of confusion and spatial disorientation in the rats. Behavioral analysis however indicated that animals were generally able to discriminate novel from familiar environments (Figure S1). While there are possible confounding factors in the behavioral assessment in the two-compartment maze because the conditions differ systematically in order, time after wake-up and environmental geometry, the data from the O-maze experiments suggest behavioral novelty detection (Figure S1A). We therefore conclude that firing rates were unaffected by novelty despite its detection by the animal.

Although novelty detection appeared to be largely intact under our experimental conditions, spatial disorientation in the novel environment immediately following wake up, together with associated emotional responses (i.e. fear, stress, anxiety), might have acted in concert with contextual novelty to account for the observed low activity of deep layer neurons. Novelty signals are inevitably associated with complex emotional responses in animals (Beerling et al., 2011) and a large variety of neuromodulatory transmitters appear to mediate these responses (Ihalainen et al., 1999; Barry and Hasselmo, 2012). Indeed the MEC receives prominent innervation from midbrain dopaminergic neurons (Björklund and Dunnett, 2007), as well as from adrenergic (Fallon et al., 1978), and serotoninergic neurons (Bobillier et al., 1975). However, the fact that low levels of activity were also observed under conditions of reduced stress, such as in familiar environments, indicates that sparse firing is a constitutive property of deep layers of MEC. Further work will elucidate whether sparse firing is inherited from upstream regions (i.e., CA1), or whether it is a result of local synaptic interactions.

## Author contributions

Andrea Burgalossi performed the experiments, Michael Brecht supervised the experiments. Moritz von Heimendahl wrote MATLAB analysis code, Andrea Burgalossi and Moritz von Heimendahl analyzed data. All authors wrote the manuscript.

### Conflict of interest statement

The authors declare that the research was conducted in the absence of any commercial or financial relationships that could be construed as a potential conflict of interest.

## Supplementary material

Figure S1 **Rats' behavior indicates novelty detection. (A)** Per-rat mean speed during exploration of either a novel or a familiar O-maze. **(B)** As in **(A)**, but for rats in the two-compartment maze. **(C)** For rats in the novel environment of the two-compartment maze, share of time spent in the central quarter of the maze. The red line indicates the expected share assuming a uniform sampling of the space by the rat. **(D)** In the two-compartment maze, share of time spent in the familiar environment after first entering it. The red line indicates indifferent choice (50%).

Figure S2 **Distribution of neuronal firing rates in medial entorhinal cortex. (A)** Firing rate histogram for all cells (*n* = 70). To better resolve low firing rates all cells were assigned an extra virtual spike, leading to different rates for a recording without observed spikes of, e.g., 10 s (rate 1·10^−1^ Hz) as opposed to 10 min (rate 0.2·10^−2^ Hz) (see Materials and Methods). **(B)** Firing rate histograms as in **(A)**, but for superficial (light blue; *n* = 33) and deep layer cells (dark blue, *n* = 37).
