# Supplementary figures and images for: Deep layer neurons in the rat medial entorhinal cortex fire sparsely irrespective of spatial novelty
Source: Front Neural Circuits. 2014 Jul 11;8:74. doi: 10.3389/fncir.2014.00074 (PMC4092364; doi:10.3389/fncir.2014.00074)

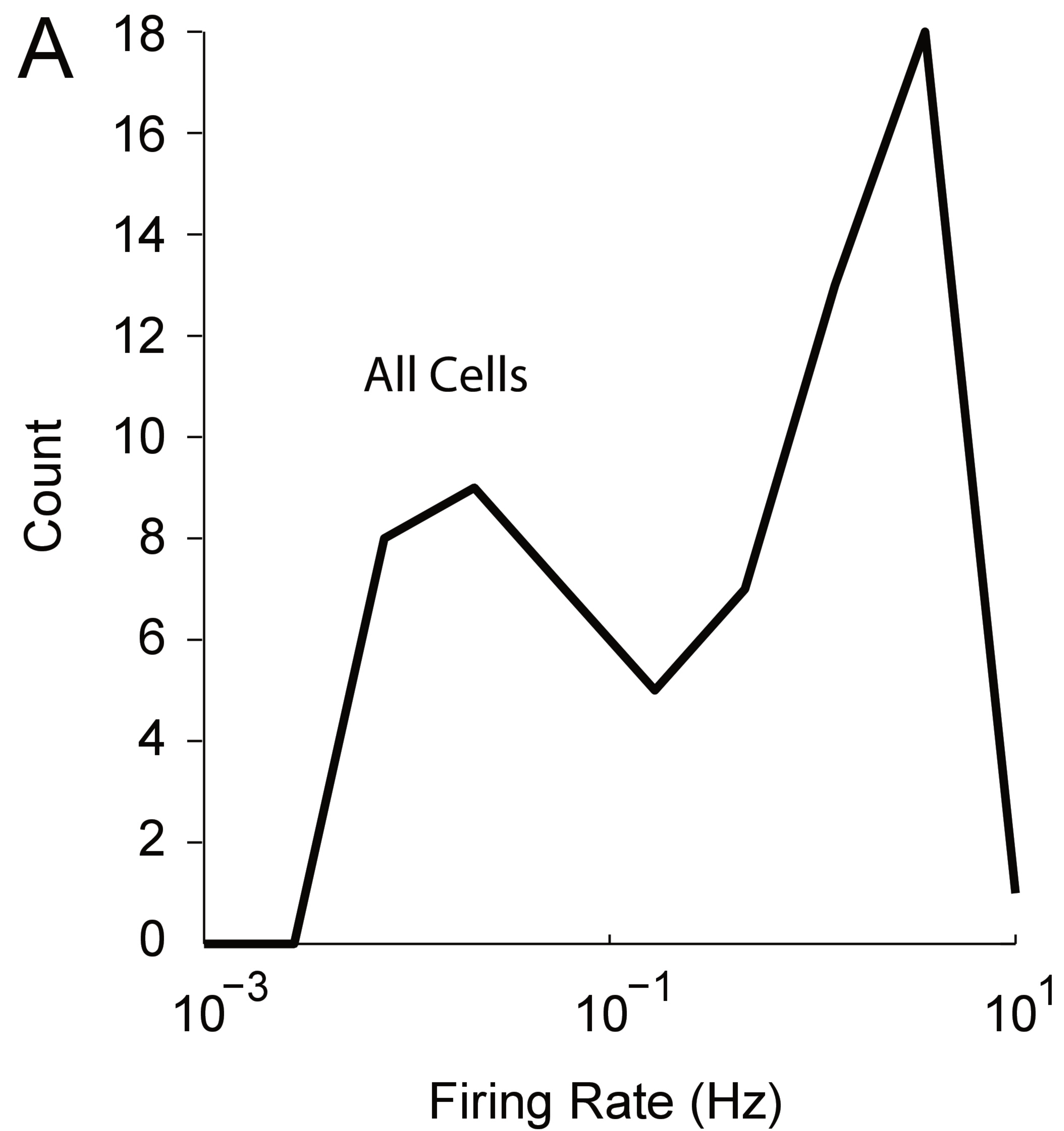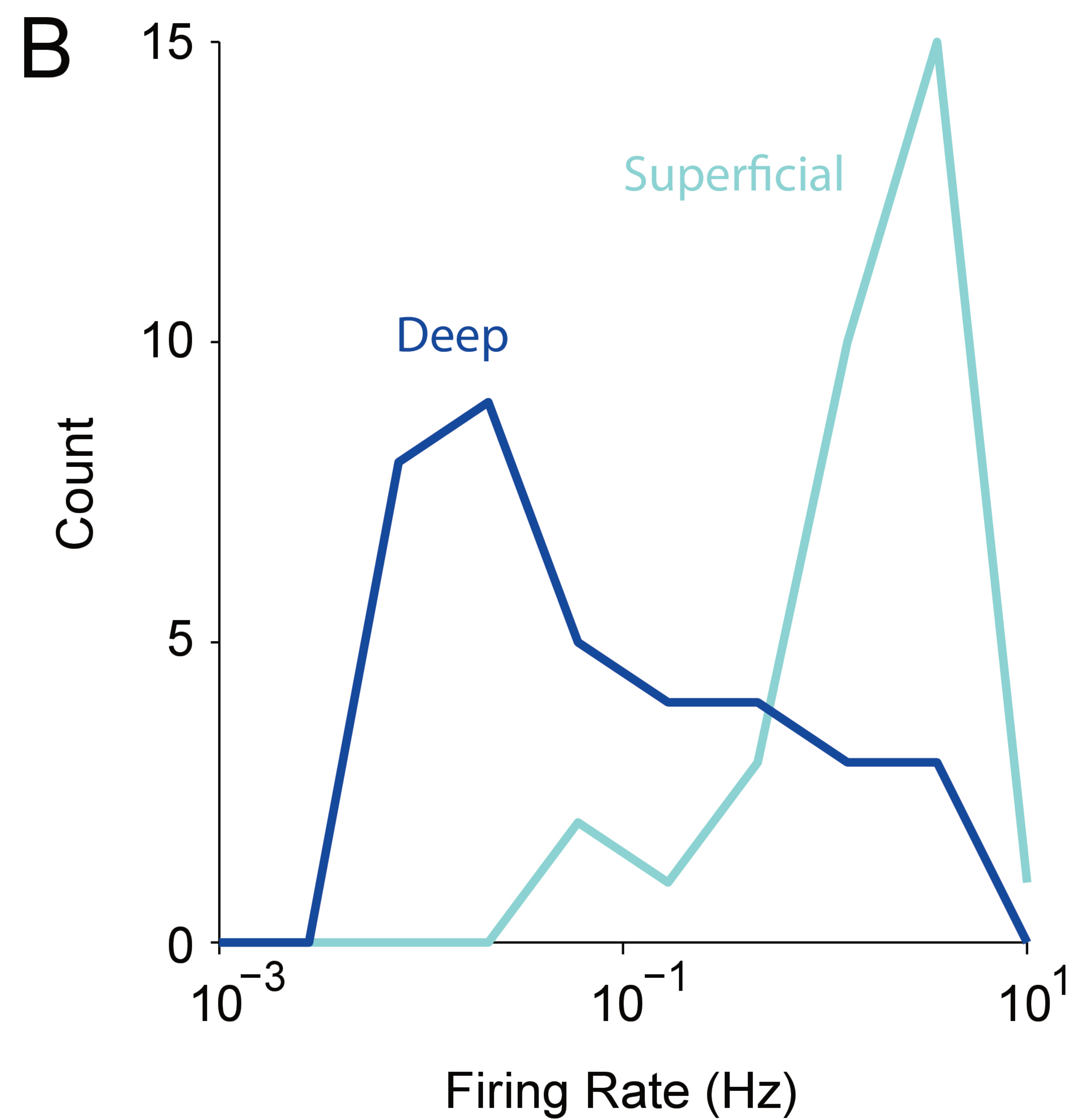

Supplement: Figure S2 — Distribution of neuronal firing rates in medial entorhinal cortex. (A) Firing rate histogram for all cells (n = 70). To better resolve low firing rates all cells were assigned an extra virtual spike, leading to different rates for a recording without observed spikes of, e.g., 10 s (rate 1·10−1 Hz) as opposed to 10 min (rate 0.2·10−2 Hz) (see Materials and Methods). (B) Firing rate histograms as in (A), but for superficial (light blue; n = 33) and deep layer cells (dark blue, n = 37). [file Presentation2.PDF]
