# Supplementary material for: Deep layer neurons in the rat medial entorhinal cortex fire sparsely irrespective of spatial novelty
Source: Front Neural Circuits. 2014 Jul 11;8:74. doi: 10.3389/fncir.2014.00074 (PMC4092364; doi:10.3389/fncir.2014.00074)
Supplement: Figure S1 — Rats' behavior indicates novelty detection. (A) Per-rat mean speed during exploration of either a novel or a familiar O-maze. (B) As in (A), but for rats in the two-compartment maze. (C) For rats in the novel environment of the two-compartment maze, share of time spent in the central quarter of the maze. The red line indicates the expected share assuming a uniform sampling of the space by the rat. (D) In the two-compartment maze, share of time spent in the familiar environment after first entering it. The red line indicates indifferent choice (50%). [file Presentation1.PDF]

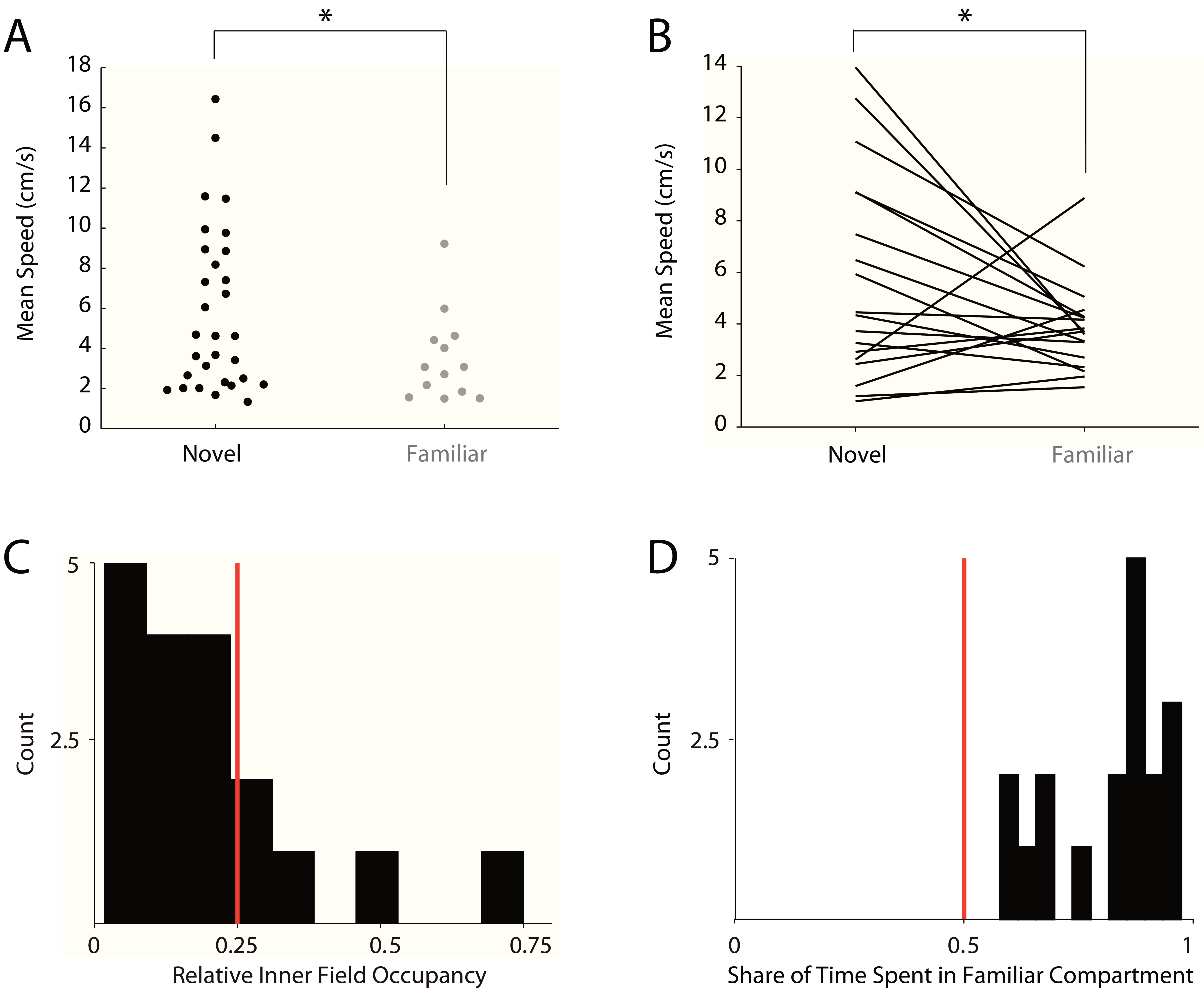
A
*
18
16
14
12
10
8
6
4
2
0
Mean Speed (cm/s)
Novel
Familiar
B
*
14
12
10
8
6
4
2
0
Mean Speed (cm/s)
Novel
Familiar
C
5
2.5
Count
0
0.25
0.5
0.75
Relative Inner Field Occupancy
D
5
2.5
Count
0
0.5
1
Share of Time Spent in Familiar Compartment
